# Temporal trends in cause-specific mortality among individuals with newly diagnosed atrial fibrillation in the Framingham Heart Study

**DOI:** 10.1186/s12916-021-02037-x

**Published:** 2021-07-29

**Authors:** Jelena Kornej, Qiuxi Huang, Sarah R. Preis, Steven A. Lubitz, Darae Ko, Joanne M. Murabito, Emelia J. Benjamin, Ludovic Trinquart

**Affiliations:** 1 Preventive Medicine and Section of Cardiovascular Medicine, Department of Medicine, Boston University School of Medicine, Boston, MA USA; 2 Boston University’s and National Heart, Lung, and Blood Institute’s Framingham Heart Study, Framingham, MA USA; 3 Department of Biostatistics, Boston University School of Public Health, 801 Massachusetts Avenue, Boston, MA 02139 USA; 4 Cardiovascular Research Center, Massachusetts General Hospital, Boston, USA; 5 Cardiac Arrhythmia Service, Division of Cardiology, Massachusetts General Hospital, Boston, USA; 6 Section of General Internal Medicine, Department of Medicine, Boston University School of Medicine, Boston, MA USA; 7 Department of Epidemiology, Boston University School of Public Health, Boston, MA USA

**Keywords:** Atrial fibrillation, Death, Temporal trends, Cardiovascular mortality, Epidemiology

## Abstract

**Background:**

All-cause mortality following atrial fibrillation (AF) has decreased over time. Data regarding temporal trends in causes of death among individuals with AF are scarce. The aim of our study was to analyze temporal trends in cause-specific mortality and predictors for cardiovascular (CVD) and non-CVD deaths among participants with incident AF in the Framingham Heart Study.

**Methods:**

We categorized all newly diagnosed AF cases according to age at AF diagnosis (< 70, 70 to < 80, and ≥ 80 years) and epoch of AF diagnosis (< 1990, 1990–2002, and ≥ 2003). We followed participants until death or the last follow-up. We categorized death causes into CVD, non-CVD, and unknown causes. For each age group, we tested for trends in the cumulative incidence of cause-specific death across epochs. We fit multivariable Fine-Gray models to assess subdistribution hazard ratios (HR) between clinical risk factors at AF diagnosis and cause-specific mortality.

**Results:**

We included 2125 newly diagnosed AF cases (mean age 75.5 years, 47.8% women). During a median follow-up of 4.8 years, 1657 individuals with AF died. There was evidence of decreasing CVD mortality among AF cases diagnosed < 70 years and 70 to < 80 years (p_trend_ < 0.001) but not ≥ 80 years (p = 0.76). Among the cases diagnosed < 70 years, the cumulative incidence of CVD death at 75 years was 67.7% in epoch 1 and 13.9% in epoch 3; among those 70 to < 80 years, the incidence at 85 years was 58.9% in epoch 1 and 18.9% in epoch 3. Advancing age (HR per 1 SD increase 6.33, 95% CI 5.44 to 7.37), prior heart failure (HR 1.49, 95% CI 1.14–1.94), and prior myocardial infarction (HR 1.44, 95% CI 1.15–1.80) were associated with increased rate of CVD death.

**Conclusions:**

In this community-based cohort, CVD mortality among AF cases decreased over time. Most deaths in individuals with AF are no longer CVD-related, regardless of age at AF diagnosis.

**Supplementary Information:**

The online version contains supplementary material available at 10.1186/s12916-021-02037-x.

## Background

A trial fibrillation (AF) is the most common sustained cardiac arrhythmia in clinical routine and affected about 59.7 million individuals globally in 2019 [1]. The lifetime risk of AF is about one in three among White people and one in five among Black people [2–4]. AF is projected to affect ~ 12–16 million people in the USA by 2050 [5] and ~ 18 million people in Europe by 2060 [6].

AF is associated with an increased risk of stroke and systemic embolism, dementia, and heart failure leading to an increased rate of premature death [7, 8]. Previous studies reported that short-term and long-term survival among individuals with AF has improved over time [9–11]. However, data on temporal trends in cause-specific mortality among individuals with AF are sparse.

For the last decades, approaches of AF management have changed drastically. Antiarrhythmic medication, catheter ablation, and anticoagulation with novel anticoagulants are guidelines that indicated management in the appropriate patients with AF [7]. Changes in therapies over time may translate into changes in the distribution of causes of death. Secondary analyses of randomized trials and observational studies in contemporary anticoagulated AF populations, all with medium-term follow-up durations, reported that the majority of deaths were not related to stroke, but due to other cardiovascular causes, primarily heart failure [12–16]. Moreover, little is known about the predictors of cause-specific mortalities among AF patients. Assessing the distribution of death causes after AF diagnosis and identifying predictors of cause-specific mortalities in AF patients may help develop targeted interventions and reduce mortality.

Using data from the Framingham Heart Study (FHS), we aimed to assess the temporal trends in cause-specific mortality among participants diagnosed with AF and to assess predictors of death from cardiovascular diseases (CVD) and non-CVD causes among individuals with AF.

## Methods

We used data from participants with incident AF in the FHS. Because AF is diagnosed over a wide range of ages and because the distribution of causes of death may differ according to age at AF diagnosis, we classified AF cases into three pre-specified groups of age at AF diagnosis: < 70 years, 70 to < 80 years, and ≥ 80 years. In each group of age at AF diagnosis, we examined the trends in cause-specific mortality over three pre-specified epochs: AF diagnosis < 1990, between 1990 and 2002, and ≥ 2003. We also assessed the predictors of cause-specific death.

### Data sources

We analyzed data from the FHS Original Cohort, which enrolled 5209 participants between 1948 and 1953; the Offspring Cohort with 5124 participants enrolled in 1971–1975; Omni 1 Cohort with 506 participants in 1994–1998; Third-Generation Cohort with 4095 participants enrolled in 2002–2005; New Offspring Spouse Cohort with 171 participants in 2003–2005; and Omni 2 Cohort with 410 participants enrolled in 2003–2005 [17–19]. The FHS protocols are reviewed by the Institutional Review Board of Boston University Medical Center, and all participants provided informed consent.

### Participants and atrial fibrillation definition

We included all FHS participants with newly diagnosed AF. We did not include participants with prevalent AF at enrollment (n = 141). We combined atrial flutter with AF. At least two FHS cardiologists adjudicated AF or atrial flutter that was observed by electrocardiograms from FHS research examinations, participants’ medical appointments, or hospital records. FHS personnel contacted hospitals and medical clinics to collect the participants’ medical records. We further defined secondary AF as AF associated with an acute reversible precipitant at the time of diagnosis, including acute myocardial infarction (within 30 days), acute cardiothoracic surgery (within 30 days), thyrotoxicosis, acute alcohol intoxication, acute infection, or acute pulmonary pathology [20].

### Clinical characteristics of atrial fibrillation cases

In descriptive analyses, we summarized the distribution of age at AF diagnosis, sex, and the following clinical characteristics: body mass index (BMI), systolic and diastolic blood pressure (BP), hypertension treatment, diabetes mellitus, current smoking, medium-severe alcohol consumption (> 7 drinks/week in women; > 14 drinks/week in men), history of heart failure, history of myocardial infarction, history of stroke or transient ischemic attack, and history of cancer. The majority of AF cases were detected outside of FHS in-person examinations. As a consequence, we collected the clinical characteristics at the closest FHS examination prior to AF diagnosis, except history of heart failure, history of myocardial infarction, history of stroke or transient ischemic attack, and history of cancer which were always assessed at the time of AF diagnosis. We also assessed if participants received anticoagulation or antiplatelet therapy at discharge at the time of AF diagnosis.

### Death review and classification

We followed the participants until their death, last FHS examination or medical contact, or end of follow-up (December 31, 2016). Death reviews were performed using information from hospitalization and emergency department records, imaging and laboratory reports, physicians’ notes, death certificates, and autopsy/medical examiners’ reports [10]. In selected cases, family members, caregivers, or eyewitnesses who were present at the time of death were interviewed to collect additional details. A panel of three FHS physicians conducted death reviews. At least two of the three members were required to concur on the cause of death. The underlying cause of death was defined as the disease or injury leading directly to death. When more than one event could have contributed to death, the degree to which the conditions contributed established the primacy of the final cause of death (Additional file 1: Supplementary Text).

Based on a detailed evaluation of all available data sources, the Framingham Endpoint Review Committee classified the underlying cause of death into coronary heart disease (CHD), stroke, other CVD disease, cancer, non-CVD/non-cancer cause, or unknown. For our primary analysis, we classified the causes of death into CVD death (CHD, stroke, and other CVD), non-CVD death, or unknown cause.

### Temporal trends in cause-specific mortality

We categorized AF cases into nine groups according to the epoch of AF diagnosis and the individuals’ age at the time of AF diagnosis (< 70 years, 70 years to < 80 years, and ≥ 80 years). For each group, we estimated the cumulative incidence of each cause of death, while accounting for competing risks of other causes of death by using the Aalen-Johansen estimator [21]. We used age as the time scale. For each age group, we tested for linear trend over epochs in cause-specific cumulative incidence by using Fine-Gray subdistribution hazard models with a sandwich variance estimator [22, 23]. In these analyses, we coded the epochs by using orthogonal polynomial trend contrasts. We reported the cumulative incidence estimates at landmark ages: 75 years for AF diagnosis < 70 years, 85 years for AF diagnosis between 70 and < 80 years, and 95 years for AF diagnosis ≥ 80 years. In exploratory analyses, we examined the cumulative incidence of deaths from CHD, stroke, and other CVD causes. In a sensitivity analysis, we repeated the main analyses among AF cases without prior cancer, considering that non-CVD mortality rates are higher among individuals with a history of cancer. In subgroup analyses, we repeated the main analyses for secondary and non-secondary AF cases. We also tested if the trends differed between secondary and non-secondary AF.

### Predictors of cause-specific mortality

To identify predictors of cause-specific deaths, we considered only incident AF cases who attended an FHS in-person exam within 10 years prior to AF diagnosis. We fitted multivariable Fine-Gray subdistribution hazard models with sandwich variance estimator to assess the associations between clinical covariates and cause-specific mortality. We report subdistribution hazard ratios (sHR) and associated 95% confidence intervals. Our primary model was adjusted for age at AF diagnosis, sex, systolic and diastolic BP, BMI, current smoking, elevated alcohol consumption, diabetes, hypertension treatment, history of heart failure, history of myocardial infarction, history of stroke or transient ischemic attack, history of cancer, secondary vs. non-secondary AF, anticoagulation at AF diagnosis, antiplatelet therapy at AF diagnosis, and epoch of AF diagnosis [24]. In a secondary analysis, we further adjusted for estimated glomerular filtration rate (eGFR) < 60 mL/min/1.73 m^2^ in the subset of AF cases with available creatinine; we examined the serum creatinine values calibrated to standardized creatinine values to calculate eGFR with the Chronic Kidney Disease Epidemiology Collaboration (CKD-EPI) equation [25, 26]. We did not perform variable selection. To account for missing values in some covariates, we used multiple imputations [27]. We created 30 imputed datasets and combined estimates with Rubin’s rules. We also reported the complete case analysis. In a sensitivity analysis, we discarded individuals who died within 30 days of AF diagnosis, to remove the influence of conditions with high case-fatality rates.

Data management and statistical analyses were performed by SAS version 9.4 (SAS Institute, Cary, NC) and R version 3.6.1 (R Foundation for Statistical Computing, Vienna, Austria). We considered a two-sided *P* value < 0.05 statistically significant.

## Results

### Temporal trends in characteristics of AF cases

We identified 2125 individuals with incident AF, including 1087 (51.1%) from the Original cohort, 939 (44.2%) from the Offspring cohort, 10 (0.5%) from the New Offspring Spouse cohort, 58 (2.7%) from the Third-Generation cohort, 23 (1.1%) from the Omni 1 cohort, and 8 (0.4%) from the Omni 2 cohort. The median time from enrollment in FHS to AF diagnosis was 37.8 years in the Original cohort, 31.4 years in the Offspring cohort, 4.1 years in the New Offspring Spouse cohort, 7.6 years in the Third-Generation cohort, 9.5 years in the Omni 1 cohort, and 5.4 years in the Omni 2 cohort. The clinical characteristics of the study sample are presented in Table 1. AF was diagnosed before 1990 for 683 (32.1%) participants, between 1990 and 2002 for 711 (33.5%) participants, and in 2003 or after for 731 (34.4%) participants. AF cases diagnosed during epoch 3 were older, had higher mean BMI, and were more likely to receive antihypertensive treatment, oral anticoagulants, and antiplatelet treatment and to have diabetes, as compared to AF cases diagnosed during epoch 1. Moreover, AF cases diagnosed during epoch 3 were less likely to be current smokers and to have prior heart failure and prior myocardial infarction, as compared to AF cases diagnosed during epoch 1 (Table 1). Characteristics of individuals with AF stratified by age at AF diagnosis are presented in Additional file 1: Supplementary Tables 1-3.

**Table 1:** Characteristics of 2125 incident AF cases according to year at diagnosis

	AF diagnosis, < 1990, n = 683	AF diagnosis, 1990-2002, n = 711	AF diagnosis, ≥ 2003, n = 731
Age at diagnosis, years	73.1 ± 10.8	77.0 ± 10.9	76.3 ± 11.9
Female	327 (47.9)	350 (49.2)	338 (46.2)
Systolic BP, mmHg	146 ± 24	141 ± 23	134 ± 20
Diastolic BP, mmHg	79 ± 13	74 ± 12	72 ± 11
Body mass index, kg/m^2^	26.6 ± 4.5	27.9 ± 5.5	29.0 ± 5.7
Current smoker	155 (23.9)	106 (15.0)	77 (10.5)
Elevated alcohol consumption*	129 (23.5)	146 (21.3)	131 (18.2)
Diabetes mellitus	68 (12.5)	94 (17.1)	133 (20.6)
Hypertension treatment	267 (40.0)	369 (52.9)	427 (58.7)
Prior heart failure	169 (24.7)	155 (21.8)	119 (16.3)
Prior myocardial infarction	155 (22.7)	174 (24.5)	117 (16.0)
Prior stroke or transient ischemic attack	102 (14.9)	113 (15.9)	101 (13.8)
Prior cancer	113 (16.5)	194 (27.3)	270 (36.9)
Secondary AF	250 (36.6)	306 (43.0)	295 (40.4)
Anticoagulation at diagnosis	55 (8.1)	205 (28.8)	295 (40.4)
Antiplatelet therapy at diagnosis	75 (11.0)	229 (32.2)	383 (52.4)

Values are mean ± standard deviation or n (%)

*BP* blood pressure

*Elevated alcohol consumption is defined as > 14 drinks/week for men and > 7 drinks/week for women. Percentages of missing values in each epoch: BMI = 9.2%, 11.8%, and 10.1%; SBP = 0.3%, 2.5%, and 0%; DBP = 0.3%, 2.7%, and 0%; smoking = 5.0%, 0.6%, and 0%; alcohol consumption = 19.8%, 3.4%, and 1.5%; diabetes = 20.1%, 22.8%, and 11.8%; and hypertension treatment = 2.3%, 2.0%, and 0.4%, respectively

### Temporal trends in cause-specific mortality

During a median follow-up of 4.8 years (25th–75th percentiles 1.3–9.6 years), 1657 individuals with AF died, with 658 (39.7%) deaths of CVD causes, 804 (48.5%) of non-CVD causes, and 195 (11.8%) with unknown causes; 468 AF cases were still alive at the end of follow-up.

**Fig. 1:**
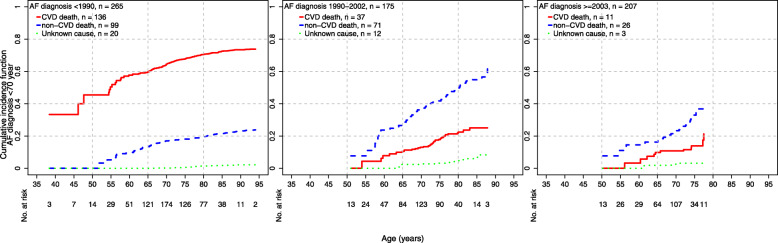
Cumulative incidences of cause-specific mortality among AF cases < 70 years and epoch of diagnosis. In each group defined by age at diagnosis and epoch of diagnosis, the plot shows age as the time scale on the horizontal axis and the cumulative incidence for each cause of death on the vertical axis. Each cumulative incidence curve starts with the youngest case at the time of diagnosis

**Fig. 2:**
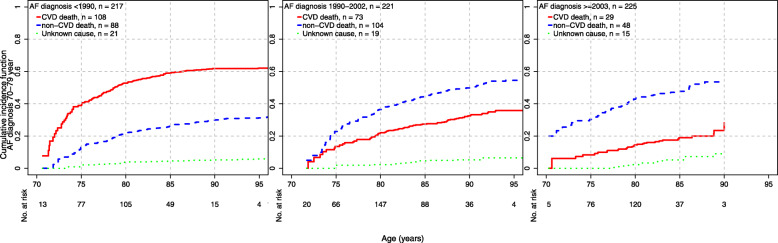
Cumulative incidences of cause-specific mortality among AF cases 70–79 years and epoch of diagnosis

**Fig. 3:**
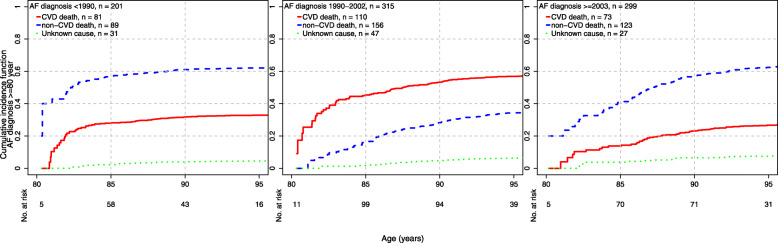
Cumulative incidences of cause-specific mortality among AF cases ≥ 80 years and epoch of diagnosis

The cumulative incidences of cause-specific mortality across epochs and age at AF diagnosis are presented in Figs. 1, 2, and 3. There was evidence of a decrease over time in CVD mortality among individuals diagnosed < 70 years (p_trend_ < 0.001) and between 70 and < 80 years (p_trend_ < 0.001, Table 2). Among AF cases diagnosed < 70 years, the cumulative incidence of CVD death at age 75 years was 67.7% in epoch 1, 17.9% in epoch 2, and 13.9% in epoch 3. Among AF cases diagnosed between 70 years and < 80 years, the cumulative incidence of CVD death at age 85 years was 58.9% in epoch 1, 27.4% in epoch 2, and 18.9% in epoch 3. Finally, among AF cases diagnosed ≥ 80 years, there was no evidence of change in CVD mortality over epochs (p_trend_ = 0.76).

**Table 2:** Cumulative incidence of cause-specific mortality at landmark ages

Age at diagnosis	AF diagnosis, < 1990, N = 683	AF diagnosis, 1990-2002, N = 711	AF diagnosis, ≥ 2003, N = 731	P_**trend**_ value
< 70 years (cumulative incidence at age **75 years**)				
CVD death	67.7 (40.8, 91.3)	17.9 (10.2, 30.4)	13.9 (6.9, 26.8)	< 0.001
Non-CVD death	18.2 (6.9, 42.9)	41.8 (29.5, 56.7)	32.9 (20.6, 50.0)	0.07
Unknown death	0.6 (0.2, 2.1)	3.2 (1.3, 8.0)	3.2 (0.9, 11.1)	0.74
Between 70 and < 80 years (cumulative incidence at age **85 years**)				
CVD death	58.9 (48.0, 70.2)	27.4 (18.8, 39.1)	18.9 (9.0, 37.5)	< 0.001
Non-CVD death	26.5 (18.7, 36.9)	44.5 (34.4, 55.9)	47.8 (27.0, 73.8)	0.30
Unknown death	4.4 (2.2, 8.6)	4.7 (2.3, 9.2)	5.2 (2.5, 10.8)	0.86
≥ 80 years (cumulative incidence at age **95 years**)				
CVD death	32.9 (14.4, 63.9)	56.9 (42.8, 71.9)	26.7 (14.9, 45.0)	0.76
Non-CVD death	62.1 (36.2, 87.7)	34.3 (23.3, 48.4)	62.5 (44.5, 80.5)	0.002
Unknown death	4.5 (1.7, 11.5)	6.4 (3.5, 11.4)	7.5 (3.5, 15.7)	0.83

In each group according to age at AF diagnosis, we tested for linear trends over epochs of AF diagnosis in the cumulative incidence function for cause-specific death

In the exploratory analysis separating CVD death causes (death from CHD, stroke, or other CVD causes), there was evidence of a decrease in cumulative incidence of CHD deaths among AF cases diagnosed between 70 and < 80 years (p_trend_ < 0.001) and ≥ 80 years (p_trend_ = 0.04), but not among those diagnosed < 70 years (p_trend_ = 0.07) (Additional file 1: Supplementary Table 4). Among the cases diagnosed 70 to < 80 years, the incidence of CHD death at 85 years was 28.2% in epoch 1, 5.9% in epoch 2, and 4.5% in epoch 3; among those ≥ 80 years, the incidence of CHD death at 95 years was 19.4% in epoch 1, 34.8% in epoch 2, and 4.0% in epoch 3 (Additional file 1: Supplementary Table 4; Supplementary Figure 1). Also, there was evidence of a decrease in stroke death among AF cases diagnosed between 70 and < 80 years (p_trend_ < 0.001) but not the 2 other age groups (p_trend_ = 0.15 and p_trend_ = 0.94). In AF cases diagnosed between 70 and < 80 years, the incidence of stroke death at 85 years was 13.8% in epoch 1, 7.5% in epoch 2, and 1.5% in epoch 3.

The cumulative incidence of non-CVD death increased among AF cases diagnosed < 70 years, from 18.2% in epoch 1 to 41.8% in epoch 2 and 32.9% in epoch 3, although the trend did not reach statistical significance (p_trend_ = 0.07; Table 2). Among the AF cases diagnosed ≥ 80 years, it dropped from 62.1% in epoch 1 to 34.3% in epoch 2 but increased back to 62.5% in epoch 3 (p_trend_ = 0.002). There was no evidence of change for cases diagnosed between 70 and < 80 years (p_trend_ = 0.30). Finally, there was no evidence of the trend over time in deaths from unknown causes.

In all, 577 (27%) AF cases had prior cancer at AF diagnosis. The median time from cancer diagnosis to AF diagnosis was 7.7 years [Q1–Q3 2.6–14.6 years]. In the sensitivity analysis excluding AF cases with prior cancer, the results were overall consistent. In particular, CVD mortality decreased over time among individuals diagnosed < 70 years (p_trend_ < 0.001) and between 70 and < 80 years (p_trend_ < 0.001, Additional file 1: Supplementary Figure 2).

### Temporal trends in secondary and non-secondary AF

There were 851 (40.1%) individuals with secondary AF at diagnosis. At the end of follow-up, 243 had died because of CVD, 374 because of non-CVD, and 68 because of unknown causes, while 166 were still alive. Among the 1274 individuals with non-secondary AF, 415 died of CVD, 430 of non-CVD, and 127 of unknown causes, while 302 were still alive at the end of follow-up.

We present the temporal trends in cause-specific mortality for secondary and non-secondary AF in Additional file 1: Supplementary Table 5 and Supplementary Figures 3 and 4. There was no evidence of differences in the patterns and temporal trends between secondary and non-secondary AF for CVD and non-CVD death, except for a decrease over time of CVD death among secondary AF cases diagnosed ≥ 80 years but in their non-secondary AF counterparts. In addition, there was evidence of an increase over time in death from unknown causes in individuals with secondary AF but not in non-secondary AF.

### Predictors of cause-specific mortality

We included 1920 AF cases who attended an exam within the 10 years prior to diagnosis in the predictors of cause-specific mortality analyses; we excluded 205 AF cases because they did not attend an exam within 10 years. The clinical characteristics of the 1920 AF cases are presented in Additional file 1: Supplementary Table 6. In the multivariable analysis, higher mean age (sHR 6.33, 95% CI 5.44–7.37), prior heart failure (sHR 1.49, 95% CI 1.14–1.94), and prior myocardial infarction (sHR 1.44, 95% CI 1.15–1.80) were associated with increased rate of CVD death (Table 3). Moreover, advanced age (sHR 7.61, 95% CI 6.54–8.86), current smoking (sHR 1.37, 95% CI 1.11–1.69), prior cancer (sHR 1.94, 95% CI 1.65–2.28), and secondary AF (sHR 1.42, 95% CI 1.22–1.66) were associated with increased rate of non-CVD death.

**Table 3:** Multivariable Fine-Gray model for cause-specific mortality among AF cases

AF cases, n = 1,920	CVD death (n = 599)		Non-CVD death (n = 722)	
	sHR	*P value*	sHR	*P value*
Age at diagnosis, years	6.33 (5.44, 7.37)	< 0.001	7.61 (6.54, 8.86)	< 0.001
Female	0.96 (0.80, 1.14)	0.60	1.06 (0.91, 1.25)	0.44
Systolic BP, mmHg	1.24 (0.81, 1.88)	0.32	0.91 (0.75, 1.10)	0.32
Diastolic BP, mmHg	1.05 (0.95, 1.16)	0.32	1.05 (0.95, 1.16)	0.32
Body mass index, kg/m^2^	0.98 (0.90, 1.08)	0.71	0.94 (0.86, 1.02)	0.14
Current smoker	0.95 (0.74, 1.22)	0.67	1.37 (1.11, 1.69)	0.004
Elevated alcohol consumption	0.86 (0.70, 1.06)	0.16	1.06 (0.88, 1.27)	0.55
Diabetes	1.18 (0.93, 1.48)	0.17	0.88 (0.70, 1.10)	0.26
Hypertension treatment	1.18 (0.99, 1.41)	0.06	0.86 (0.74, 1.01)	0.06
Prior heart failure	1.49 (1.14, 1.94)	0.003	0.70 (0.51, 0.96)	0.02
Prior myocardial infarction	1.44 (1.15, 1.80)	0.002	0.89 (0.71, 1.12)	0.32
Prior stroke or transient ischemic attack	1.27 (0.99, 1.64)	0.06	0.96 (0.75, 1.23)	0.75
Prior cancer	0.56 (0.46, 0.70)	< 0.001	1.94 (1.65, 2.28)	< 0.001
Secondary AF	0.92 (0.78, 1.10)	0.36	1.42 (1.22, 1.66)	< 0.001
Anticoagulation at diagnosis	1.00 (0.80, 1.24)	0.98	0.88 (0.72, 1.07)	0.20
Antiplatelet therapy at diagnosis	0.71 (0.57, 0.88)	0.002	0.99 (0.82, 1.18)	0.88
Epoch				
1990–2002 vs. < 1990	0.69 (0.56, 0.85)	< 0.001	1.22 (1.01, 1.47)	0.04
≥ 2003 vs. < 1990	0.74 (0.57, 0.97)	0.03	1.18 (0.92, 1.52)	0.18

Elevated alcohol consumption is defined as > 14 drinks/week for men and > 7 drinks/week for women

For continuous covariates, the sHR corresponds to 1 standard deviation increase in the covariate (1 SD for age = 11.5 years, BMI 5.3 kg/m^2^, systolic blood pressure 22.9 mmHg, diastolic blood pressure 12.2 mmHg)

This analysis included 1920 AF cases who attended an exam within 10 years prior to diagnosis. After a median follow-up of 4.8 years, 1493 AF cases had died (599 CVD death, 722 non-CVD death, 172 unknown cause) while 427 were still alive

*sHR* subdistribution hazard ratio, *BP* blood pressure

The complete case analysis, based on 1422 patients with variables within 10 years, yielded similar results (Additional file 1: Supplementary Table 7). In the sensitivity analysis excluding individuals who died within 30 days of AF (n = 186), the results were similar as well (Additional file 1: Supplementary Table 8).

In the multivariable model in the subset of 1691 participants with available eGFR data, the results were similar (Additional file 1: Supplementary Table 9). The models were not substantively changed, and eGFR was not significantly associated with CVD death or non-CVD death.

## Discussion

In our analysis of the FHS, we observed decreasing trends over time in CVD death among participants with AF diagnosed before 70 years and between 70 and < 80 years. Regardless of age at AF diagnosis, the majority of deaths were not related to CVD in the most recent epoch, i.e., AF diagnosed in 2003 or after. The decrease in CVD death was driven by a decrease in CHD and stroke deaths. In addition, when comparing CVD and non-CVD death trends between individuals with secondary and non-secondary AF, we found that CVD death decreased over time among secondary AF cases diagnosed ≥ 80 years but not among their non-secondary AF counterparts. Finally, in adjusted models, advancing age, prior heart failure, and prior myocardial infarction were predictors of CVD death among individuals with AF.

### Interpretation of our findings

AF management has improved dramatically since the first participants were included in the FHS. Both pharmacological treatments and advanced individualized strategies have changed. Warfarin and direct oral anticoagulants are associated with decreased mortality [28]. In randomized trials, warfarin reduced mortality by over 25% compared to placebo [29], whereas direct oral anticoagulants decreased mortality by 10% compared to vitamin K antagonists [28]. Finally, several studies have highlighted the importance of AF prevention through lifestyle changes [30, 31]. Improvements in prevention, detection, and management are paralleled by increased awareness of AF among clinicians and patients, and growing amounts of scientific data have led to frequent updates in AF management guidelines [7, 8].

Our results regarding the decrease in CVD death over time may be explained by significant improvements in the treatment of CVD and cardiometabolic risk factors, and improved treatments of CVD and of AF treatment [32]. Among AF cases in our study, the mean blood pressure levels declined, while the proportion of individuals receiving hypertension treatment increased. We also observed that the prevalence of current smoking and history of heart failure decreased over time. All factors can probably explain the decline in CVD mortality [33, 34]. Recent advances in pharmacological and device implementation as a standard therapy for heart failure with reduced ejection fraction might further contribute to reductions of CVD mortality over time. Finally, active implementation of oral anticoagulation for stroke prevention over three decades ago led to a significant reduction of thromboembolic events associated with AF [35] explaining the relevant decrease of CVD death in our study. Conversely, we observed an increase in metabolic risk; in particular, the mean BMI levels and the prevalence of diabetes increased over time among AF cases. This rise is paradoxical when considering the decline in CVD death [36]. Other factors not quantified in our study, in particular, social determinants of health, may contribute to these patterns. However, identifying the causes of the decline in CVD mortality rates is beyond the scope of our analyses.

### Comparison with other studies

Several contemporary randomized trials of direct oral anticoagulants and observational studies described the distribution of causes of death among AF patients (Additional file 1: Supplementary Table 10) [12–14, 16, 37]. Previous studies had median follow-up durations ranging from about 1 to 3.3 years. In our current analysis, the median follow-up is 4.8 years, and we were able to assess the temporal changes in death causes over more than three decades.

With regard to CVD deaths, several randomized trials and large registries reported the relevance of heart failure as a major death cause [12–16]. In the RE-LY Registry, sudden death and heart failure resulted in about one-third of all deaths, while 8% of deaths resulted from stroke [37]. In our study, prevalent heart failure was an important risk factor for CVD mortality. Furthermore, we found that heart failure was associated with a 1.49-fold increase in the rate of CVD death. These results suggest the necessity to control and improve heart failure management in individuals with AF. At the same time, the evidence of temporal decrease of CVD death is consistent with a positive impact of heart failure management strategies during the last decades.

In our study, we also analyzed if cause-specific mortality differed among individuals with a reversible AF precipitant. Despite the strong association between acute comorbidity (especially myocardial infarction, thoracic or cardiac surgery, acute alcohol consumption) and increased AF recurrence risk during follow-up, in a prior study, no differences were reported in mortality rates [20]. In our current analysis, we confirm previous findings as we found overall similar patterns of CVD and non-CVD death among individuals with secondary and non-secondary AF.

With regard to non-CVD death, the proportion of non-CVD death causes in previous randomized trials and observational studies ranged from 30 to 49% with malignancies and infections as the main causes of death [12–16]. Our results are consistent with previous studies, with 48.5% of deaths from non-CVD causes. In our analysis, advancing age was associated with an 8-fold increase in non-CVD death rate per ~ 11 years (standard deviation) increase in age, while current smoking was associated with a 37% increase in non-CVD mortality rate.

### Study limitations

Several limitations could influence the interpretation of our results. First, the study population was largely of European ancestry, and many participants lived in the New England area. Thus, the generalizability of the results to other races/ethnicities or other regions and countries is limited. Second, we combined AF and atrial flutter, and we did not distinguish between AF subtypes (paroxysmal, persistent, or permanent). In previous studies, permanent AF was associated with increased rate of heart failure but with a decreased rate of coronary event, as compared to paroxysmal AF [38], and recurrent and sustained AF were associated with increased rate of all-cause mortality [39]. Moreover, there may be misclassification of AF, which is often clinically unrecognized and may change over time. Similarly, we cannot rule out the misclassification of death causes particularly among older adults (e.g., sudden death or death without witness). Third, we had a relatively small sample size with a small number of deaths for some age groups and epochs, or when looking at CHD, stroke, and other CVD mortality. It may limit the statistical power to detect the trends, and large p values do not indicate evidence of absence of trends. Fourth, multivariable analyses may have been influenced by unmeasured and/or uncontrolled confounding. Fifth, although we included anticoagulation status at the time of diagnosis in the multivariable analyses, we did not consider this variable as a predictor, because of selection biases in who received anticoagulation and because we had incomplete data on anticoagulation status in follow-up. Also, we did not incorporate other factors that could explain the trends in CVD and non-CVD deaths, for example, anemia [14, 16] or treatments of AF-associated comorbidities such as diabetes and heart failure. Finally, we did not analyze whether the decline in CVD death among individuals with AF is similar, faster, or slower as compared to the decline in CVD death in the overall FHS population and in individuals without AF.

## Conclusions

In conclusion, based on the Framingham Heart Study data, CVD mortality among AF cases decreased over time, with a decrease in CHD and stroke deaths. Since 2003, most deaths are not CVD-related, regardless of age at the time of AF diagnosis. Older age at AF diagnosis, history of myocardial infarction, history of heart failure, and history of stroke or transient ischemic attack were associated with increased rate of CVD death.

## Supplementary Information

**Additional file 1.** Supplementary Methods and Results. Methods used for classification of causes of death and results from subgroup and sensitivity analyses.

## Data Availability

Participant-level data from the Framingham Heart Study are available at the database of Genotypes and Phenotypes (https://www.ncbi.nlm.nih.gov/gap/) and BioLINCC (https://biolincc.nhlbi.nih.gov/home/).

## Abbreviations

AF: Atrial fibrillation
BMI: Body mass index
BP: Blood pressure
CHD: Coronary heart disease
CKD-EPI: Chronic Kidney Disease Epidemiology Collaboration
CVD: Cardiovascular
eGFR: Estimated glomerular filtration rate
FHS: Framingham Heart Study
HR: Hazard ratio
sHR: Subdistribution hazard ratio
