# Supplementary material for: Temporal trends in cause-specific mortality among individuals with newly diagnosed atrial fibrillation in the Framingham Heart Study
Source: BMC Med. 2021 Jul 29;19:170. doi: 10.1186/s12916-021-02037-x (PMC8320026; doi:10.1186/s12916-021-02037-x)

**Temporal trends in cause-specific mortality among individuals with atrial fibrillation in the Framingham Heart Study – Supplementary Material**

Contents

[Supplementary Text. Classification of causes of death 2](#_Toc72508729)

[Supplementary Table 1. Characteristics of AF cases diagnosed <70 years according to epoch of AF diagnosis 4](#_Toc72508730)

[Supplementary Table 2. Characteristics of AF cases diagnosed between 70 and <80 years according to epoch of AF diagnosis 4](#_Toc72508731)

[Supplementary Table 3. Characteristics of AF cases diagnosed ≥80 years according to epoch of AF diagnosis 5](#_Toc72508732)

[Supplementary Table 4. Cumulative incidence of CHD, stroke, and other CVD mortality at landmark ages 5](#_Toc72508733)

[Supplementary Table 5. Trend test p-values among secondary and non-secondary AF cases 6](#_Toc72508734)

[Supplementary Table 6. Characteristics of 1,920 AF cases who attended an FHS exam within 10 years prior to AF diagnosis 6](#_Toc72508735)

[Supplementary Table 7. Multivariable Fine-Gray model for predicting cause-specific mortality among AF individuals with complete data 7](#_Toc72508736)

[Supplementary Table 8. Sensitivity analysis to the multivariable Fine-Gray model, excluding AF cases who died within 30 days of AF diagnosis 8](#_Toc72508737)

[Supplementary Table 9. Multivariable Fine-Gray model including glomerular filtration rate 9](#_Toc72508738)

[Supplementary Table 10. Distribution of causes of death among patients with AF from previous observational and randomized studies 10](#_Toc72508739)

[Supplementary Figure 1. Cumulative incidences of CHD death, stroke death, and other CVD death according to age and epoch of diagnosis. 11](#_Toc72508740)

[Supplementary Figure 2. Cumulative incidences of cause-specific mortality among AF cases without prior cancer according to age and epoch of diagnosis. 12](#_Toc72508741)

[Supplementary Figure 3. Cumulative incidences of cause-specific mortality among secondary AF cases according to age and epoch of diagnosis. 13](#_Toc72508742)

[Supplementary Figure 4. Cumulative incidences of cause-specific mortality among non-secondary AF cases according to age and epoch of diagnosis. 14](#_Toc72508743)

## Supplementary Text. Classification of causes of death

The Framingham Endpoint Review Committee classified the underlying cause of each death into coronary heart disease (CHD), stroke, other CVD disease, cancer, non-CVD/non-cancer cause, or unknown.

CHD

Participants were diagnosed as having developed CHD if, upon review of the case, the Framingham Endpoint Review Committee agreed on one of the following definite manifestations of CHD: myocardial infarction (MI), coronary insufficiency syndrome, or angina pectoris. Each manifestation is defined as follows.

*Myocardial Infarction.* Recent or acute MI is designated when there were at least two of three findings: 1) symptoms indicative of ischemia; 2) changes in biomarkers of myocardial necrosis; 3) serial changes in the electrocardiograms indicating the evolution of an infarction, including the loss of initial QRS potentials (that is, development of “pathologic” Q-waves of 0.04 second duration or greater). An old or remote MI is considered to be present when the electrocardiogram shows a stable pattern including a pathologic Q-wave of 0.04 second or greater or loss of initial QRS potential R-wave in those leads in which this would not be expected to occur. Also, an interim unrecognized MI is indicated when changes from a previous tracing show development of loss of R-wave potential or appearance of pathologic Q-waves not otherwise explained, in persons in whom neither the patient nor his physician considered the possibility of MI. If the patient was asymptomatic for chest pain or upper abdominal pain during the interval at which the unrecognized MI occurred, the event is classified as silent, unrecognized. More weight is given to this finding if a T-wave abnormality is also associated with Q-wave abnormality. An autopsy report showing an acute, new, or recent infarction of the myocardium is accepted as evidence of an incident MI. Because it is not possible to date an old infarction found on autopsy, such evidence is not used in the clinical diagnosis of a new event, unless there was an interim clinical event suspected of being an infarction.

*Coronary Insufficiency syndrome.* It was designated when a history of prolonged ischemic chest pain (> 15 minutes duration) was accompanied by transient ischemic S-T segment and T-wave abnormality in the electrocardiographic tracing but not accompanied by development of Q-wave abnormality or by serum enzyme changes characteristic of myocardial necrosis.

*Angina Pectoris.* Brief recurrent chest discomfort of up to 15 minutes duration, precipitated by exertion or emotion and relieved by rest or by nitroglycerine is regarded as angina pectoris (AP) if two physicians interviewing the subject at a Framingham clinic visit or the Framingham Endpoint Review Committee, upon review of medical records, agree that this condition was definitely present. This diagnosis is based solely on evaluation of subjective manifestations. Abnormality of the resting or exercise electrocardiogram is not required for this diagnosis.

CHD death

Death from coronary heart disease was diagnosed as either sudden or nonsudden.

*Non-sudden death from CHD* if the terminal episode lasted longer than one hour, if the available information implies that the cause of death was probably CHD, and if no other cause can be ascribed. In making this diagnosis, the review panel uses prior clinical information as well as information concerning the final illness.

*Sudden death from CHD* if a participant, apparently well, was observed to have died within a few minutes (operationally documented as under one hour) from onset of symptoms and if the cause of death cannot reasonably be attributed on the basis of the full clinical information and the information concerning death to some potentially lethal disease other than CHD.

Stroke

The diagnosis is based on the occurrence of a clinically evident stroke documented by clinical records reviewed by at least two neurologists. Stroke is defined as the sudden or rapid onset of a focal neurologic deficit persisting for greater than 24 hours. Stroke is further categorized into infarction or hemorrhage.

*Ischemic Stroke.* A diagnosis of cerebral embolism is made when an established source for embolus including atrial fibrillation, rheumatic heart disease with mitral stenosis, recent myocardial infarction, bacterial endocarditis or other known source is determined. A clinical course consistent with embolic infarction or evidence of other systemic embolism may be present. Symptoms are usually rapid with maximal severity at onset.

*Hemorrhagic Stroke.* The diagnosis of subarachnoid hemorrhage is based on a history suggestive of this process such as abrupt onset headache, with or without change in the level of consciousness, and signs of meningeal irritation with or without other localizing neurological deficits. Intracerebral hemorrhage is diagnosed clinically by the occurrence of abrupt focal neurologic deficit, often with altered level of consciousness and symptoms of increased intracranial pressure. Hemorrhages are confirmed by imaging.

*Antherothrombotic brain infarction* is defined as the sudden onset of a focal neurologic deficit lasting longer than 24 hours, in the absence of:

1) known source of embolism (atrial fibrillation, rheumatic heart disease with mitral stenosis, myocardial infarction within preceding six months, bacterial endocarditis);

2) intracranial hemorrhage (intracerebral or subarachnoid);

3) known hypercoagulable states;

4) other disease processes causing focal neurologic deficits (brain tumor, subdural hematoma, hypoglycemia).

Confirmatory imaging supports the diagnosis.

Stroke Death

Death attributed to stroke is designated when a documented focal neurologic deficit of greater than 24 hours duration preceded death and was responsible for the fatality.

The Framingham Endpoint Review Committee defined the immediate cause of death as well as contributing causes of death. Our analyses are based on immediate causes of death. When participants with prior MI died of heart failure, there are 3 possible scenarios: 1) The individual had MI and as a consequence developed acute heart failure; the immediate cause of death was identified as MI, as it was the main cause of clinical presentation, which was decompensated by heart failure – contributing cause of death; 2) The individual had MI many years/months prior to developing chronic heart failure and died because of its decompensation. In this case, the immediate cause of death was heart failure, categorized as other CVD, and CHD/MI was a contributing cause of death; 3) The individual had both MI and heart failure, but died from another cause, for example infection or cancer. In this case, both MI and heart failure would be contributing causes only.

## Supplementary Table 1. Characteristics of AF cases diagnosed <70 years according to epoch of AF diagnosis

|  |  | **Diagnosis**  **<1990** | **Diagnosis**  **1990-2002** | **Diagnosis**  **≥2003** |
| --- | --- | --- | --- | --- |
|  |  | n=265 | n=175 | n=207 |
| Age at diagnosis, years |  | 62.3±6.7 | 61.8±6.4 | 61.3±7.6 |
| Females |  | 97 (36.6) | 43 (24.6) | 64 (30.9) |
| Systolic BP, mmHg |  | 142±25 | 133±23 | 126±15 |
| Diastolic BP, mmHg |  | 83±13 | 79±12 | 77±9 |
| Body mass index, kg/m^2^ |  | 27.2±4.5 | 30.2±6.4 | 30.2±5.8 |
| Current smoker |  | 98 (38.3) | 45 (25.9) | 36 (17.4) |
| Elevated alcohol consumption |  | 65 (32.3) | 42 (24.0) | 49 (24.0) |
| Diabetes |  | 21 (10.3) | 32 (18.8) | 37 (18.4) |
| Hypertension treatment |  | 80 (30.5) | 79 (45.4) | 81 (39.1) |
| Prior heart failure |  | 61 (23.0) | 25 (14.3) | 14 (6.8) |
| Prior myocardial infarction |  | 67 (25.3) | 48 (27.4) | 22 (10.6) |
| Prior stroke or transient ischemic attack |  | 32 (12.1) | 15 (8.6) | 14 (6.8) |
| Prior cancer |  | 31 (11.7) | 35 (20) | 52 (25.1) |
| Secondary AF |  | 99 (37.4) | 84 (48.0) | 71 (34.3) |
| Anticoagulation at diagnosis |  | 34 (12.8) | 53 (30.3) | 89 (43.0) |
| Antiplatelet therapy at diagnosis |  | 26 (9.8) | 64 (36.6) | 107 (51.7) |

## Supplementary Table 2. Characteristics of AF cases diagnosed between 70 and <80 years according to epoch of AF diagnosis

|  |  | **Diagnosis**  **<1990** | **Diagnosis**  **1990-2002** | **Diagnosis**  **≥2003** |
| --- | --- | --- | --- | --- |
|  |  | n=217 | n=221 | n=225 |
| Age at diagnosis, years |  | 74.9±2.8 | 75.7±2.7 | 75.5±3.0 |
| Females |  | 102 (47.0) | 108 (48.9) | 104 (46.2) |
| Systolic BP, mmHg |  | 146±23 | 141±21 | 134±21 |
| Diastolic BP, mmHg |  | 79±12 | 73±11 | 72±11 |
| Body mass index, kg/m^2^ |  | 26.8±4.6 | 27.6±5.0 | 29.0±5.9 |
| Current smoker |  | 42 (20.4) | 37 (16.8) | 26 (11.6) |
| Elevated alcohol consumption |  | 43 (25.6) | 55 (25.0) | 42 (18.7) |
| Diabetes |  | 30 (17.2) | 41 (20.5) | 51 (23.7) |
| Hypertension treatment |  | 91 (43.3) | 113 (52.6) | 137 (61.4) |
| Prior heart failure |  | 52 (24.0) | 45 (20.4) | 31 (13.8) |
| Prior myocardial infarction |  | 48 (22.1) | 50 (22.6) | 45 (20.0) |
| Prior stroke or transient ischemic attack |  | 25 (11.5) | 35 (15.8) | 34 (15.1) |
| Prior cancer |  | 44 (20.3) | 64 (29.0) | 93 (41.3) |
| Secondary AF |  | 77 (35.5) | 100 (45.3) | 103 (45.8) |
| Anticoagulation at diagnosis |  | 11 (5.1) | 73 (33.0) | 102 (45.3) |
| Antiplatelet therapy at diagnosis |  | 24 (11.1) | 76 (34.4) | 125 (55.6) |

## Supplementary Table 3. Characteristics of AF cases diagnosed ≥80 years according to epoch of AF diagnosis

|  |  | **Diagnosis**  **<1990** | **Diagnosis**  **1990-2002** | **Diagnosis**  **≥2003** |
| --- | --- | --- | --- | --- |
|  |  | n=201 | n=315 | n=299 |
| Age at diagnosis, years |  | 85.5±4.0 | 86.3±4.6 | 87.3±4.9 |
| Females |  | 128 (63.7) | 199 (63.2) | 170 (56.9) |
| Systolic BP, mmHg |  | 151±23 | 145±23 | 140±21 |
| Diastolic BP, mmHg |  | 75±12 | 72±12 | 69±11 |
| Body mass index, kg/m^2^ |  | 25.2±3.8 | 26.6±4.6 | 28.1±5.3 |
| Current smoker |  | 15 (8.0) | 24 (7.7) | 15 (5.0) |
| Elevated alcohol consumption |  | 21 (11.7) | 49 (16.8) | 40 (13.8) |
| Diabetes |  | 17 (10.1) | 21 (11.7) | 45 (19.7) |
| Hypertension treatment |  | 96 (49.2) | 177 (57.5) | 209 (70.1) |
| Prior heart failure |  | 56 (27.9) | 85 (27.0) | 74 (24.8) |
| Prior myocardial infarction |  | 40 (19.9) | 76 (24.1) | 50 (16.7) |
| Prior stroke or transient ischemic attack |  | 45 (22.4) | 63 (20.0) | 53 (17.7) |
| Prior cancer |  | 38 (18.9) | 95 (30.2) | 125 (41.8) |
| Secondary AF |  | 74 (36.8) | 122 (38.7) | 121 (40.5) |
| Anticoagulation at diagnosis |  | 10 (5.0) | 79 (25.1) | 104 (34.8) |
| Antiplatelet therapy at diagnosis |  | 25 (12.4) | 89 (28.3) | 151 (50.5) |

##

## Supplementary Table 4. Cumulative incidence of CHD, stroke, and other CVD mortality at landmark ages

| Age at diagnosis | **Diagnosis**  **<1990** | **Diagnosis**  **1990-2002** | **Diagnosis**  **≥2003** | **P_trend_** |
| --- | --- | --- | --- | --- |
| <70 years (cumulative incidence at age 75 years) | | |  |  |
| CHD death | 13.4 (5.1, 32.7) | 8.3 (2.9, 22.6) | 8.3 (3.5, 18.7) | 0.07 |
| Stroke death | 6.9 (1.3, 33.1) | 0.8 (0.2, 3.3) | 4.4 (1.0, 19.1) | 0.15 |
| Other CVD death | 47.5 (16.2, 90.4) | 8.8 (4.4, 17.0) | 1.2 (0.2, 8.2) | 0.002 |
| Between 70 and <80 years (cumulative incidence at age 85 years) | | |  |  |
| CHD death | 28.2 (16.5, 45.7) | 5.9 (3.4, 10.1) | 4.5 (1.8, 10.9) | <0.001 |
| Stroke death | 13.8 (7.7, 24.3) | 7.5 (2.2, 24.2) | 1.5 (0.5, 4.4) | <0.001 |
| Other CVD death | 16.9 (10.6, 26.4) | 14.0 (8.7, 22.3) | 13.0 (4.8, 32.5) | 0.37 |
| ≥80 years (cumulative incidence at age 95 years) | | |  |  |
| CHD death | 19.4 (7.9, 43.2) | 34.8 (19.1, 57.9) | 4.0 (1.5, 11.0) | 0.04 |
| Stroke death | 5.6 (2.0, 15.1) | 10.9 (5.0, 23.0) | 4.7 (1.7, 12.2) | 0.94 |
| Other CVD death | 7.9 (2.5, 23.4) | 11.2 (6.5, 19.2) | 18.0 (9.1, 33.7) | 0.14 |

## Supplementary Table 5. Trend test p-values among secondary and non-secondary AF cases

| **Secondary AF** |  |  |  |
| --- | --- | --- | --- |
| Age at AF diagnosis | CVD death | Non-CVD death | Unknown |
| <70 year | <0.001 | 0.75 | 0.72 |
| Between 70 and <80 years | 0.007 | 0.54 | 0.48 |
| ≥80 years | 0.04 | 0.05 | 0.001 |
| **Non-secondary AF** |  |  |  |
| Age at AF diagnosis | CVD death | Non-CVD death | Unknown |
| <70 year | <0.001 | 0.05 | 0.46 |
| Between 70 and <80 year | <0.001 | 0.46 | 0.12 |
| ≥80 years | 0.16 | 0.02 | 0.16 |
| **Interaction** |  |  |  |
| Age at AF diagnosis | CVD death | Non-CVD death | Unknown |
| <70 year | 0.81 | 0.19 | 0.49 |
| Between 70 and <80 years | 0.77 | 0.80 | 0.12 |
| ≥80 years | 0.03 | 0.62 | 0.002 |

Among secondary and non-secondary AF cases, we tested for linear trends over epochs of AF diagnosis in the cumulative incidence function for cause-specific death, in each group according to age at AF diagnosis. We also tested if the linear trends were different between secondary and non-secondary AF.

## Supplementary Table 6. Characteristics of 1,920 AF cases who attended an FHS exam within 10 years prior to AF diagnosis

|  |  | **Diagnosis**  **<1990** | **Diagnosis**  **1990-2002** | **Diagnosis**  **≥2003** |
| --- | --- | --- | --- | --- |
|  |  | **n=650** | **n=634** | **n=636** |
| Age at diagnosis, years |  | 72.9±10.8 | 76.7±10.7 | 76.0±12.2 |
| Female |  | 308 (47.4) | 302 (47.6) | 290 (45.6) |
| Systolic BP, mmHg |  | 146±24 | 140±23 | 134±20 |
| Diastolic BP, mmHg |  | 79±13 | 73±12 | 71±11 |
| Body mass index, kg/m^2^ |  | 26.6±4.5 | 28.0±5.4 | 29.0±5.7 |
| Current smoker |  | 138 (22.4) | 78 (12.3) | 52 (8.2) |
| Elevated alcohol consumption |  | 124 (23.5) | 127 (20.5) | 104 (16.6) |
| Diabetes |  | 67 (12.8) | 89 (18.4) | 120 (21.4) |
| Hypertension treatment |  | 262 (41.3) | 349 (56.2) | 388 (61.3) |
| Prior heart failure |  | 156 (24.0) | 131 (20.7) | 106 (16.7) |
| Prior myocardial infarction |  | 146 (22.5) | 156 (24.6) | 108 (17.0) |
| Prior stroke or transient ischemic attack |  | 98 (15.1) | 103 (16.3) | 87 (13.7) |
| Prior cancer |  | 110 (16.9) | 180 (28.4) | 237 (37.2) |
| Secondary AF |  | 232 (35.7) | 272 (42.9) | 250 (39.3) |
| Anticoagulation at diagnosis |  | 51 (7.9) | 183 (28.9) | 266 (41.8) |
| Antiplatelet therapy at diagnosis |  | 69 (10.6) | 208 (32.8) | 340 (53.5) |

## Supplementary Table 7. Multivariable Fine-Gray model for predicting cause-specific mortality among AF individuals with complete data

| N AF cases = 1,422 | CVD death (n= 392) | | Non-CVD death (n= 521) | |
| --- | --- | --- | --- | --- |
|  | sHR | p-value | sHR | p-value |
| Age at diagnosis, years | 4.75 (3.91, 5.76) | <0.001 | 5.20 (4.39, 6.15) | <0.001 |
| Female | 0.96 (0.79, 1.17) | 0.67 | 0.98 (0.82, 1.17) | 0.81 |
| Systolic BP, mmHg | 1.11 (1.00, 1.24) | 0.05 | 1.04 (0.94, 1.16) | 0.41 |
| Diastolic BP, mmHg | 1.07 (0.96, 1.20) | 0.20 | 0.93 (0.84, 1.02) | 0.14 |
| Body mass index, kg/m^2^ | 1.00 (0.90, 1.12) | 0.93 | 0.93 (0.85, 1.03) | 0.16 |
| Current smoker | 0.79 (0.60, 1.05) | 0.11 | 1.59 (1.28, 1.98) | <0.001 |
| Elevated alcohol consumption | 0.91 (0.72, 1.15) | 0.42 | 1.04 (0.86, 1.26) | 0.67 |
| Diabetes | 1.20 (0.93, 1.55) | 0.17 | 0.90 (0.71, 1.14) | 0.37 |
| Hypertension treatment | 1.20 (0.99, 1.47) | 0.07 | 0.91 (0.77, 1.07) | 0.25 |
| Prior heart failure | 2.16 (1.72, 2.72) | <0.001 | 0.74 (0.59, 0.92) | 0.006 |
| Prior myocardial infarction MI | 1.79 (1.44, 2.23) | <0.001 | 0.62 (0.50, 0.77) | <0.001 |
| Prior stroke or transient ischemic attack | 1.75 (1.37, 2.23) | <0.001 | 0.65 (0.50, 0.84) | <0.001 |
| Prior cancer | 0.69 (0.54, 0.88) | 0.003 | 2.09 (1.75, 2.50) | <0.001 |
| Secondary AF | 0.73 (0.60, 0.89) | 0.002 | 1.61 (1.36, 1.91) | <0.001 |
| Anticoagulation at diagnosis | 0.92 (0.73, 1.18) | 0.52 | 0.92 (0.75, 1.14) | 0.46 |
| Antiplatelet therapy at diagnosis | 0.77 (0.61, 0.97) | 0.03 | 1.06 (0.88, 1.29) | 0.53 |
| Year at AF diagnosis |  |  |  |  |
| 1990-2002 vs. <1990 | 0.77 (0.61, 0.96) | 0.02 | 1.05 (0.87, 1.28) | 0.60 |
| ≥2003 vs. <1990 | 0.70 (0.52, 0.95) | 0.02 | 0.88 (0.67, 1.16) | 0.35 |

Out of 1,920 AF cases who attended exam within 10 years prior to AF diagnosis, 498 had missing values for at least one covariate while 1,422 AF cases had complete covariate data.

## Supplementary Table 8. Sensitivity analysis to the multivariable Fine-Gray model, excluding AF cases who died within 30 days of AF diagnosis

| N AF cases = 1,734 | CVD death (n=511) | | Non-CVD death (n=630) | |
| --- | --- | --- | --- | --- |
|  | sHR | p-value | sHR | p-value |
| Age at diagnosis, years | 4.44 (3.82, 5.16) | <0.001 | 5.15 (4.43, 5.98) | <0.001 |
| Female | 0.94 (0.78, 1.13) | 0.49 | 1.05 (0.88, 1.24) | 0.60 |
| Systolic BP, mmHg | 1.16 (0.87, 1.56) | 0.32 | 0.95 (0.86, 1.05) | 0.32 |
| Diastolic BP, mmHg | 1.06 (0.95, 1.18) | 0.32 | 1.05 (0.95, 1.17) | 0.32 |
| Body mass index, kg/m^2^ | 0.96 (0.87, 1.06) | 0.46 | 0.96 (0.88, 1.05) | 0.39 |
| Current smoker | 0.95 (0.72, 1.24) | 0.69 | 1.30 (1.03, 1.64) | 0.03 |
| Elevated alcohol consumption | 0.87 (0.70, 1.09) | 0.22 | 1.04 (0.86, 1.27) | 0.68 |
| Diabetes | 1.23 (0.96, 1.58) | 0.10 | 0.91 (0.71, 1.15) | 0.43 |
| Hypertension treatment | 1.25 (1.03, 1.51) | 0.02 | 0.84 (0.71, 0.99) | 0.04 |
| Prior heart failure | 1.40 (1.04, 1.88) | 0.02 | 0.78 (0.56, 1.08) | 0.14 |
| Prior myocardial infarction MI | 1.31 (1.02, 1.67) | 0.03 | 0.96 (0.76, 1.23) | 0.76 |
| Prior stroke or transient ischemic attack | 1.28 (0.97, 1.69) | 0.09 | 0.99 (0.75, 1.30) | 0.93 |
| Prior cancer | 0.62 (0.49, 0.78) | <0.001 | 1.73 (1.45, 2.07) | <0.001 |
| Secondary AF | 0.85 (0.70, 1.03) | 0.09 | 1.34 (1.13, 1.58) | <0.001 |
| Anticoagulation at diagnosis | 1.11 (0.89, 1.40) | 0.36 | 0.91 (0.74, 1.11) | 0.35 |
| Antiplatelet therapy at diagnosis | 0.79 (0.63, 0.99) | 0.04 | 1.02 (0.84, 1.24) | 0.82 |
| Year at AF diagnosis |  |  |  |  |
| 1990-2002 vs. <1990 | 0.63 (0.51, 0.79) | <0.001 | 1.29 (1.05, 1.58) | 0.02 |
| ≥2003 vs. <1990 | 0.64 (0.47, 0.86) | 0.003 | 1.10 (0.84, 1.45) | 0.50 |

To account for missing values in some covariates, we used multiple imputation. Out of 1,920 AF cases who attended exam within 10 years prior to AF diagnosis, 186 died within 30 days of AF diagnosis and were excluded from this analysis.

## Supplementary Table 9. Multivariable Fine-Gray model including glomerular filtration rate

| N AF cases = 1,691 | CVD death (n=456) | | Non-CVD death (n=657) | |
| --- | --- | --- | --- | --- |
|  | sHR | p-value | sHR | p-value |
| Age at diagnosis, years | 7.41 (6.24, 8.80) | <0.001 | 7.67 (6.47, 9.10) | <0.001 |
| Female | 0.93 (0.76, 1.14) | 0.48 | 1.11 (0.94, 1.31) | 0.24 |
| Systolic BP, mmHg | 1.14 (0.88, 1.48) | 0.32 | 0.95 (0.86, 1.05) | 0.32 |
| Diastolic BP, mmHg | 1.06 (0.95, 1.18) | 0.32 | 1.05 (0.95, 1.15) | 0.32 |
| Body mass index, kg/m2 | 1.00 (0.90, 1.11) | 0.98 | 0.93 (0.85, 1.02) | 0.15 |
| Current smoker | 0.97 (0.70, 1.33) | 0.84 | 1.40 (1.11, 1.77) | 0.004 |
| Elevated alcohol consumption | 0.82 (0.63, 1.06) | 0.13 | 1.10 (0.90, 1.34) | 0.35 |
| Diabetes | 1.12 (0.86, 1.44) | 0.40 | 0.95 (0.76, 1.20) | 0.69 |
| Hypertension treatment | 1.35 (1.10, 1.66) | 0.004 | 0.80 (0.68, 0.94) | 0.007 |
| Prior heart failure | 1.29 (0.94, 1.78) | 0.12 | 0.82 (0.58, 1.15) | 0.25 |
| Prior myocardial infarction MI | 1.48 (1.15, 1.92) | 0.003 | 0.90 (0.71, 1.15) | 0.40 |
| Prior stroke or transient ischemic attack | 1.28 (0.97, 1.69) | 0.09 | 1.00 (0.77, 1.28) | 0.97 |
| Prior cancer | 0.56 (0.45, 0.71) | <0.001 | 1.94 (1.65, 2.28) | <0.001 |
| eGFR<60 mL/min/1.73 m^2^ | 1.26 (1.00, 1.58) | 0.05 | 0.94 (0.77, 1.16) | 0.58 |
| Secondary AF | 0.98 (0.81, 1.19) | 0.85 | 1.34 (1.14, 1.58) | <0.001 |
| Anticoagulation at diagnosis | 1.05 (0.83, 1.33) | 0.66 | 0.85 (0.70, 1.04) | 0.11 |
| Antiplatelet therapy at diagnosis | 0.80 (0.63, 1.00) | 0.05 | 0.94 (0.78, 1.12) | 0.48 |
| Year at AF diagnosis |  |  |  |  |
| 1990-2002 vs. <1990 | 0.84 (0.66, 1.05) | 0.13 | 1.06 (0.87, 1.30) | 0.53 |
| ≥2003 vs. <1990 | 0.89 (0.66, 1.19) | 0.43 | 1.06 (0.83, 1.37) | 0.63 |

To account for missing values in some covariates, we used multiple imputation.

sHR: subdistribution hazard ratio; BP = blood pressure; TIA = transient ischemic attack

Elevated alcohol consumption is defined as >14 drinks/week for men and >7 drinks/week for women

For continuous variables, the sHR corresponds to a one SD increase

## Supplementary Table 10. Distribution of causes of death among patients with AF from previous observational and randomized studies

|  | GARFIELD-AF registry[12] | French cohort of unselected AF patients[13] | SAKURA  AF registry[14] | Fushimi AF Registry[16] | RE-LY Atrial fibrillation registry[37] | RE-LY, ROCKET-AF, ARISTOTLE, ENGAGE AF-TIMI trials*[39] |
| --- | --- | --- | --- | --- | --- | --- |
| Enrollment period | 2010-2013 | 2000-2010 | 2013-2015 | 2011-2015 | 2007-2011 | 2005-2010 |
| N participants | 17,162 | 8,962 | 3,237 | 4,045 | 15,400 | 71,683 |
| N deaths | 1,181 | 1,294 | 200 | 705 | 1,782 | 6,206 |
| Mean/median follow-up, years | 2.0† | 2.5 | 3.3 | 3.0 | 1.0† | 1.9 |
| Cardiovascular causes (heart failure, myocardial infarction, sudden death) | 24.3% | 32.4% | 25.0% | 16.3% | 41.4% | 46.0% |
| Ischemic stroke | 5.1% | 6.7% | 5.5%‡ | 4.8% | 8.4% | 5.7%‡ |
| Hemorrhage | 2.0% | 6.7% | 7.5% | 3.1% | 2.0% | 5.6% |
| Other vascular causes | 11.1% | 8.2% | 0% | 1.3% | 2.1% | 6.6% |
| Non-cardiovascular causes | 33.8% | 42.7% | 48.5% | 54.0% | 33.6% | 29.8% |
| Unknown | 23.7% | 3.3% | 13.5% | 20.5% | 12.5% | 6.2% |

*RE-LY (2005-2007), ROCKET-AF (2006-2009), ARISTOTLE (2006-2010), ENGAGE AF-TIMI 48 (2008-2010)

†Total reported follow-up

‡Includes systemic embolism

## Supplementary Figure 1. Cumulative incidences of CHD death, stroke death, and other CVD death according to age and epoch of diagnosis.


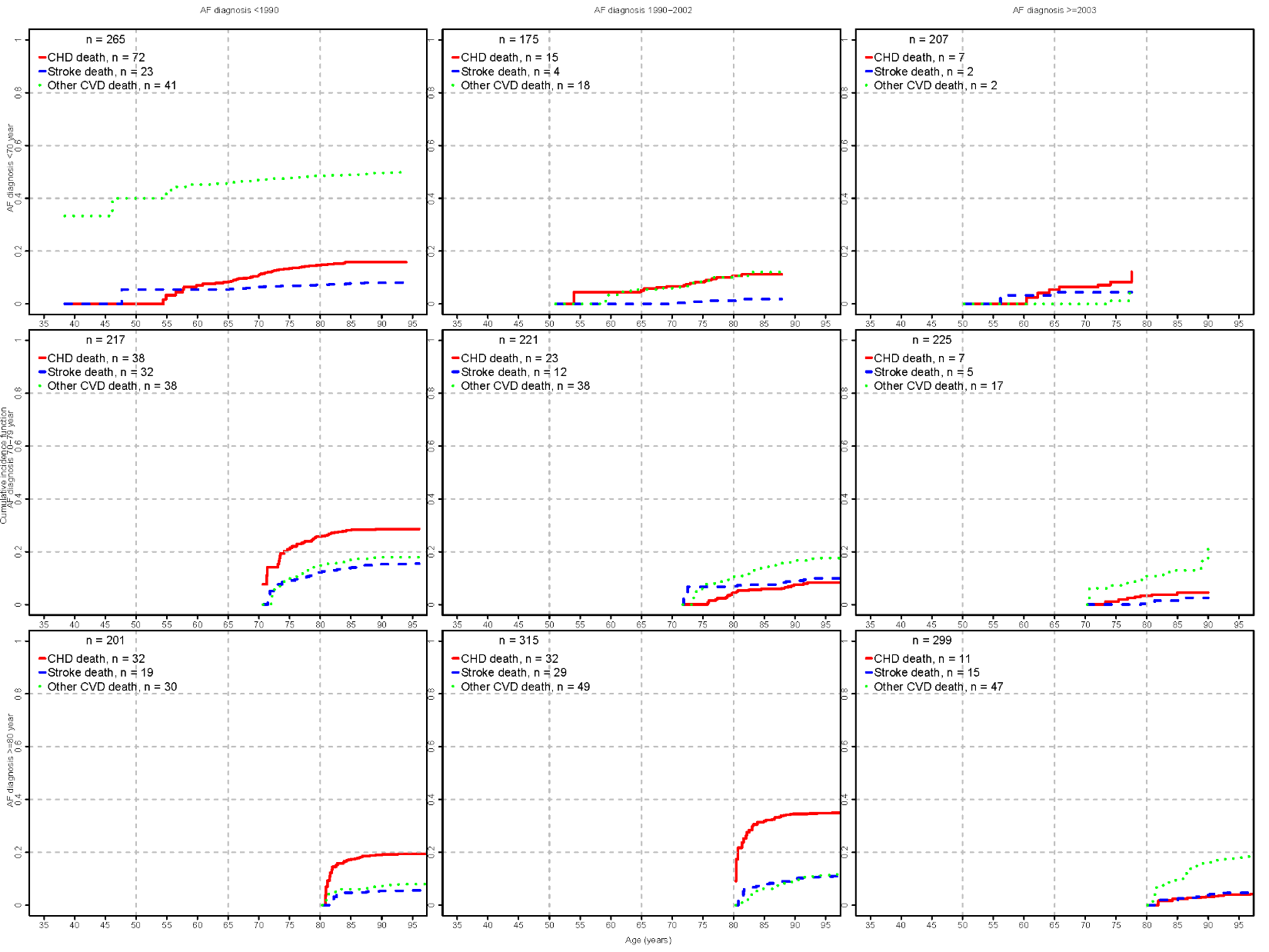


## Supplementary Figure 2. Cumulative incidences of cause-specific mortality among AF cases without prior cancer according to age and epoch of diagnosis.


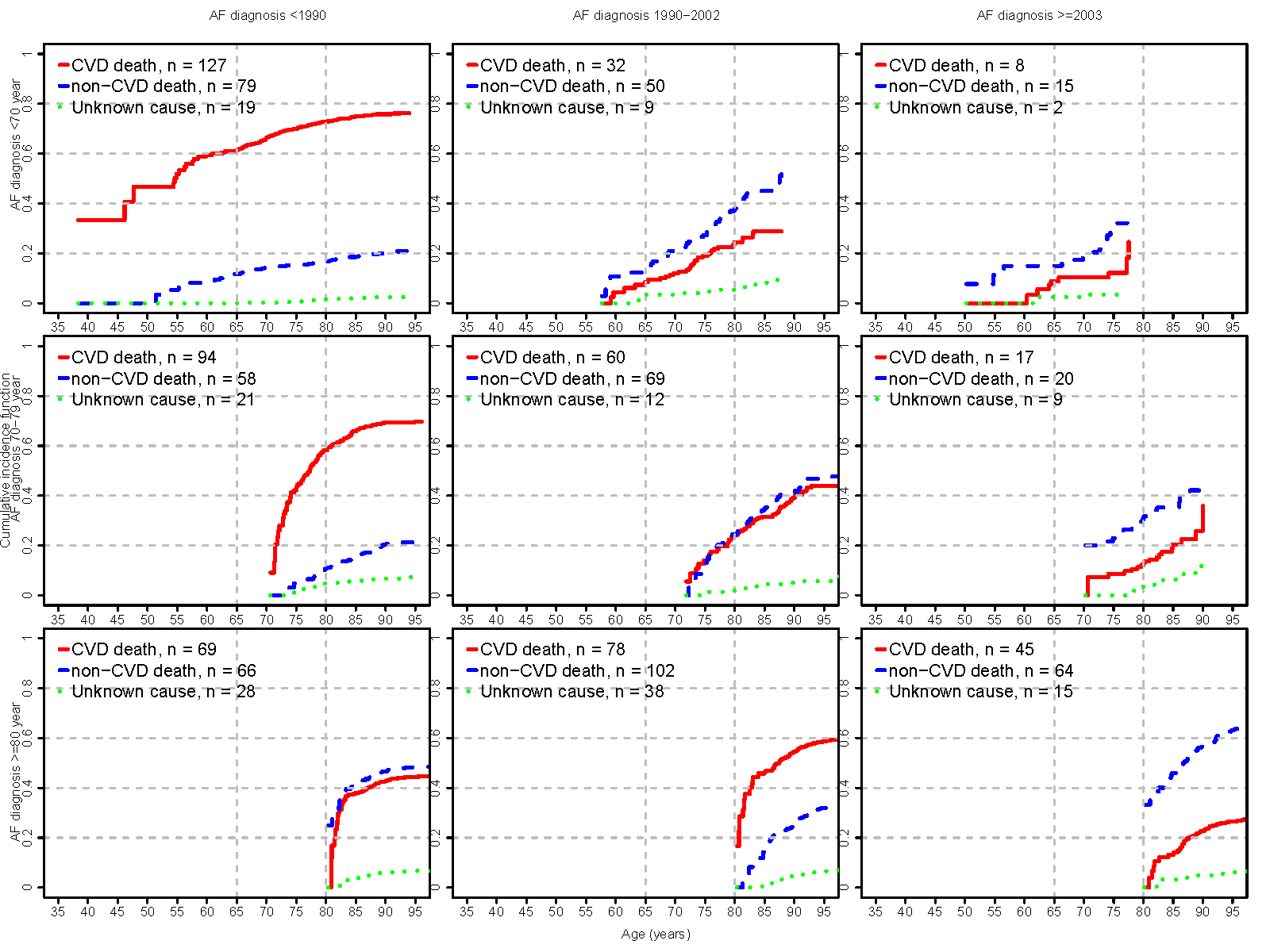


## Supplementary Figure 3. Cumulative incidences of cause-specific mortality among secondary AF cases according to age and epoch of diagnosis.


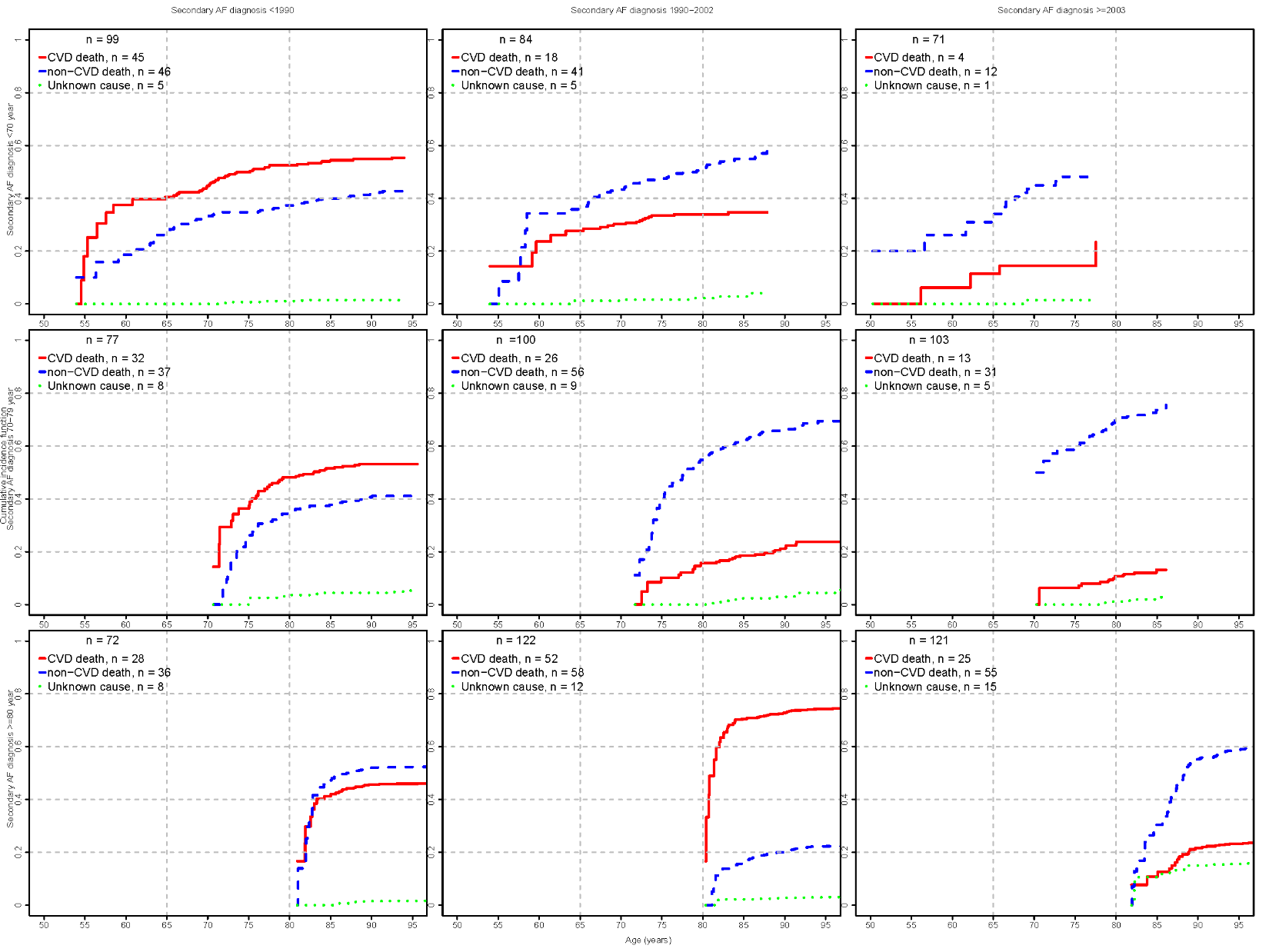
Among individuals aged ≥80 years at AF diagnosis and with AF diagnosis<1990, two participants died from non-CVD death before the 3^rd^ individual entered the risk set and as a consequence they did not contribute to the estimation of the cumulative incidence function

## Supplementary Figure 4. Cumulative incidences of cause-specific mortality among non-secondary AF cases according to age and epoch of diagnosis.


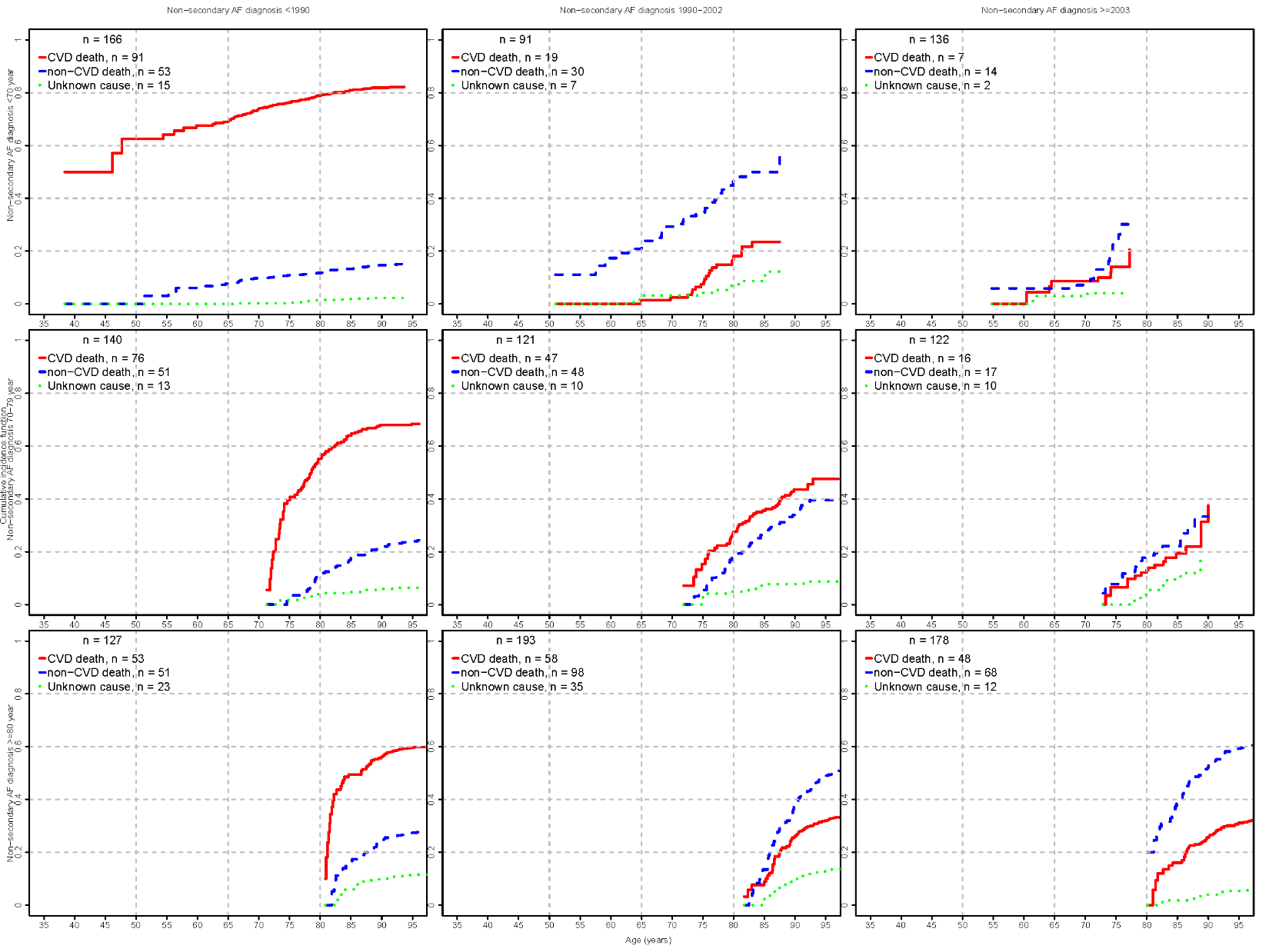

Supplement: Supplementary file 1 — Additional file 1. Supplementary Methods and Results. Methods used for classification of causes of death and results from subgroup and sensitivity analyses. [file 12916_2021_2037_MOESM1_ESM.docx]
